# PORTAF – postoperative radiotherapy of non-small cell lung cancer: accelerated versus conventional fractionation – study protocol for a randomized controlled trial

**DOI:** 10.1186/s13063-017-2346-0

**Published:** 2017-12-20

**Authors:** R. Bütof, M. Simon, S. Löck, E. G. C. Troost, S. Appold, M. Krause, M. Baumann

**Affiliations:** 1 Department of Radiotherapy and Radiation Oncology, Faculty of Medicine and University Hospital Carl Gustav Carus, Technische Universität Dresden, Dresden, Germany; 2 OncoRay – National Center for Radiation Research in Oncology, Faculty of Medicine and University Hospital Carl Gustav Carus, Technische Universität Dresden, Helmholtz-Zentrum Dresden - Rossendorf, Germany; 3 German Cancer Consortium (DKTK), partner site Dresden and German Cancer Research Center (DKFZ), Heidelberg, Germany; 4 National Center for Tumor Diseases (NCT), Partner Site Dresden, Dresden, Germany; 5 Helmholtz-Zentrum Dresden – Rossendorf, Institute of Radiooncology - OncoRay, Dresden, Germany

**Keywords:** Postoperative radiotherapy, Non-small-cell lung cancer (NSCLC), Fractionation, Acceleration, Randomized clinical trial, Phase II trial, Overall treatment time, Time factor

## Abstract

**Background:**

In early-stage non-small cell lung cancer (NSCLC) without affected lymph nodes detected at staging, surgical resection is still the mainstay of treatment. However, in patients with metastatic mediastinal lymph nodes (pN2) or non-radically resected primary tumors (R1/R2), postoperative radiotherapy (possibly combined with chemotherapy) is indicated. So far, investigations about time factors affecting postoperative radiotherapy have only examined the waiting time defined as interval between surgery and start of radiotherapy, but not the overall treatment time (OTT) itself. Conversely, results from trials on primary radio(chemo)therapy in NSCLC show that longer OTT correlates with significantly worse local tumor control and overall survival rates. This time factor of primary radio(chemo)therapy is thought to mainly be based on repopulation of surviving tumor cells between irradiation fractions. It remains to be elucidated if such an effect also occurs when patients with NSCLC are treated with postoperative radiotherapy after surgery (and chemotherapy). Our own retrospective data suggest an advantage of shorter OTT also for postoperative radiotherapy in this patient group.

**Methods/design:**

This is a multicenter, prospective randomized trial investigating whether an accelerated course of postoperative radiotherapy with photons or protons (7 fractions per week, 2 Gy fractions) improves locoregional tumor control in NSCLC patients in comparison to conventional fractionation (5 fractions per week, 2 Gy fractions). Target volumes and total radiation doses will be stratified in both treatment arms based on individual risk factors.

**Discussion:**

For the primary endpoint of the study we postulate an increase in local tumor control from 70% to 85% after 36 months. Secondary endpoints are overall survival of patients; local recurrence-free and distant metastases-free survival after 36 months; acute and late toxicity and quality of life for both treatment methods.

**Trial registration:**

ClinicalTrials.gov, NCT02189967. Registered on 22 May 2014.

**Electronic supplementary material:**

The online version of this article (doi:10.1186/s13063-017-2346-0) contains supplementary material, which is available to authorized users.

## Background

Non-small cell lung cancer (NSCLC) is one of the world’s most common malignant diseases [1, 2]. Despite the establishment of new systemic treatment options and improvement of treatment techniques, the prognosis of the patients is still poor with an average overall survival between 5.5% and 15.7% after 5 years [1, 2].

In early-stage NSCLC surgical resection is still the mainstay of treatment [2]. The role of postoperative radiotherapy (PORT) has been investigated in a number of studies and the results were summarized in two meta-analyses [3, 4]. These studies are extremely heterogeneous in terms of radiation dose, fractionation schedules and the quality of the irradiation technique. Because of this heterogeneity, clinical interpretation remains controversial. It appears clear that patients after complete surgical resection (R0) and with limited lymph node involvement (N0 or N1) do not benefit from postoperative radiation [2–4]. In contrast, there is evidence for improved local tumor control and possibly improved overall survival of patients with regionally advanced tumors (pN2) after PORT [3, 5–8]. Therefore, PORT is offered by many centers in this situation.

Based on results of randomized clinical trials and their meta-analyses [9, 10], the S3 guideline released by the German Cancer Society and the German Respiratory Society recommends the application of postoperative adjuvant chemotherapy for patients with stage III NSCLC [2], which increases survival rates after 5 years by approximately 4% [9]. The guideline also recommends consideration of PORT, which should start about 4 weeks after the end of chemotherapy [2]. The application after the end of chemotherapy leads to a delayed start of radiotherapy and a prolonged total treatment time as compared to earlier treatment concepts without chemotherapy. From a radiobiological point of view, this could lead to proliferation of tumor cells (repopulation) before the start of radiotherapy and thus to reduced locoregional control rates, at least in non- and low responders to chemotherapy. However, because of a stimulated accelerated repopulation of surviving tumor cells during chemotherapy, a reduced efficiency of PORT could arise for chemotherapy responders, too [11]. Thus far, investigations about the time factor of PORT of NSCLC patients have only examined waiting periods between surgery and the start of radiotherapy outside of sequential chemo-radiotherapy concepts. These retrospective studies have never proved a survival benefit for patients with shorter waiting periods between surgery and the start of radiotherapy [12, 13].

Randomized trials and their meta-analysis show that a longer overall treatment time (OTT) of primary radiotherapy correlates with significantly worse survival rates and local tumor control rates [14–18]. For PORT, a retrospective analysis of our own patient data indicates that such a time factor may also play an important role for NSCLC patients in the adjuvant situation [13]. Moreover, an explorative analysis based on stratification criteria of patients with a primary radiotherapy treatment within the CHARTWEL study shows that patients who received chemotherapy before radiotherapy demonstrated a significantly improved local tumor control with an HR of 0.5 after accelerated radiotherapy [16]. It remains to be elucidated if such an effect also occurs when patients are treated with PORT after chemotherapy. The aim of the present study is to investigate whether there is an improvement of local tumor control when using accelerated radiotherapy in the postoperative situation in NSCLC compared to conventional fractionation. Secondary endpoints include the evaluation of quality of life, overall survival, occurrence of distant metastases and acute and late radiation-induced side effects.

## Methods/design

This is a multicenter, prospective randomized phase II trial investigating whether accelerated postoperative radiotherapy [7 fractions per week, 2 Gray (Gy) fractions] may improve locoregional tumor control in non-small cell lung cancer (NSCLC) in comparison to conventional fractionation (5 fractions per week, 2 Gy fractions). Target volumes and total radiation doses will be stratified in both treatment arms based on individual risk factors (details see below in the Radiotherapy section). In both treatment arms irradiation may be delivered with photons or protons.

### Recruitment, randomization, and workflow

Patients will be informed about the study by the treating radiation oncologist. All patients with signed informed consent are included in the study. The following clinical examinations have to be performed before randomization: complete staging including fluorodeoxyglucose positron emission tomography/computed tomography (FDG-PET/CT) or alternatively contrast-enhanced CT chest/abdomen and bone scintigraphy; magnetic resonance imaging (MRI) of the skull only in cases of suspected brain metastases; blood analyses and postoperative pulmonary function test. For patients who received adjuvant chemotherapy, the entire staging should be repeated prior to start of radiotherapy.

**Fig. 1:**
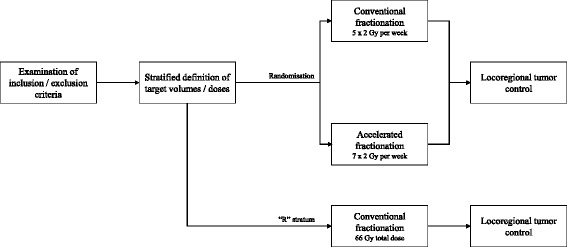
Flowchart

After finishing all obligatory pre-treatment assessments, randomization will be done electronically via RadPlanBio [19]. Patients will be stratified by the following criteria: staging with or without PET-CT; resection status R1 or R0; and the respective study center. The result of randomization will be available immediately after the patient has been registered in RadPlanBio. Patients with macroscopic tumor can be treated in stratum “R” and will receive conventionally fractionated radiation therapy to a total dose of 66 Gy according to the current clinical guideline. Figure 1 shows a flowchart of the study.

Inclusion criteria:

- histologically confirmed non-small cell lung cancer
- surgery performed with curative intention
- postoperative indication for radiotherapy (> pN1 and/or R1)
- R2 resection or recurrence postoperatively/after adjuvant chemotherapy diagnosed by the restaging imaging
- no distant metastases (M0)
- patient ≥ 18 years old
- good general health condition [Eastern Cooperative Oncology Group (ECOG) score 0 or 1]
- signed informed consent
- appropriate compliance to ensure follow-up visits
- women of childbearing age: adequate contraception

Exclusion criteria:

- histologically confirmed small cell lung cancer
- distant metastases (M1)
- patient is unable to understand reason, purpose, and side effects of this study
- previous (<5 years) or synchronous malignancy (except in situ carcinomas, basal cell carcinoma, early-stage skin cancer)
- for proton therapy: cardiac pacemaker
- previous radiotherapy in the thoracic or lower head and neck region
- pregnancy or lactation
- participation in another clinical intervention trial or not completed follow-up of a clinical intervention study (except psychological studies, supportive, or observational studies)

### Primary and secondary endpoints

The primary endpoint of this study is locoregional tumor control 36 months after start of radiotherapy comparing an accelerated irradiation schedule (7 fractions per week, 2 Gy single dose) with the standard conventional fractionation schedule (5 fractions per week, 2 Gy single dose) for POST in patients with NSCLC.

Secondary endpoints contain overall survival of patients; local recurrence-free and distant metastases-free survival 36 months after start of radiotherapy; acute and late toxicity and quality of life for both treatment methods. All primary and secondary endpoints will be compared for the photon and proton-treated patients.

Clinical follow-up examinations with additional imaging scans (FDG-PET/CT or CT thorax/abdomen) are scheduled every 3 months within the first 3 years and every 6 months within the following 2 years. During therapy and at the follow-up visits, scoring of side effects will be performed according to Common Terminology Criteria for Adverse Events (CTC-AE) 4.0. Quality of life will be assessed additionally by the European Organisation for the Research and Treatment of Cancer (EORTC) questionnaires QLQ-C30 and QLQ-LC13 before and at the end of irradiation and at each follow-up visit.

The trial design and protocol adhere to Standard Protocol items: Recommendations for Interventional Trials (SPIRIT) criteria (www.spirit-statement.org); the SPIRIT checklist and figure can be found as Additional file 1: Table S1 and Additional file 2: Figure S1.

### Biometrical design

The primary endpoint, locoregional tumor control after 36 months, will be investigated in 1:1 randomized patient cohorts treated with accelerated or conventional fractionated irradiation. Our hypothesis is an improvement of 15% after 3 years in the accelerated treatment arm (7 fractions per week) in the group of R0- or R1-resected patients; i.e., an increase of the local tumor control rate from 70% to 85%.

With accelerated irradiation, treatment time will be shortened from 33 days to 24 days if a total dose of 50 Gy is applied. If a total dose of 60 Gy is applied, treatment time will be reduced from 40 days to 29 days. On average, this means a reduction of 10 treatment days and with the assumption of gaining 0.6 Gy–0.8 Gy of dose per day (by preventing repopulation), this corresponds to an increase of the biological dose of 10–16% in the accelerated arm, depending on the prescribed total dose. Conservatively assuming a y-factor = 1 (steepness of the dose-response curve), the locoregional tumor control rate would increase by 10–16% from 70% to 80–86%.

Assuming α is 0.05, a rise of 15% can be detected with the power of 80% if 154 patients are treated in every arm. For this calculation a Cox proportional hazard model was assumed with hazard ratio 0.475, overall probability of event 22.5% and 1:1 randomization [20]. Furthermore, a drop-out rate of 10% of the participants has been used.

The “R” stratum, which contains patients with macroscopic remaining tumor or recurrences detected in pre-radiotherapeutic staging, was not included in the statistical sample size calculation.

### Radiotherapy

For radiotherapy planning purposes, all patients should undergo an FDG-PET/CT in treatment position. Alternatively, radiotherapy planning can be performed solely based on CT in treatment position. The acquisition of a four-dimensional computed tomography (4D-CT) for radiotherapy planning purposes is favored, but not mandatory for the treatment with photons. For proton therapy, the implementation of a 4D-CT is indispensable to calculate the mobility of the target volume and to consider it in the radiation treatment planning. On the basis of the planning CT, the clinical target volume (CTV) and the organs at risk are contoured. In tumors of the right lung, the CTV includes the mediastinal lymph node stations 2R, 4R, and 7 as well as the right hilum. In tumors of the left lung, the CTV includes the lymph node stations 2 L, 4 L, 5, 6, and 7 as well as the left hilum. Additionally, in tumors of the left inferior lobe levels 4R/2R are to be included. Moreover, the surgical report and surgical clips are to be considered. The CTV will subsequently be expanded to the planning target volume for photon therapy taking into account the institution’s guidelines. For proton therapy individual range uncertainties will be considered. The contouring has been standardized with a mandatory dummy run for all participating centers.

Irradiation planning will be done three dimensionally with the help of a clinically licensed treatment planning system. Every patient undergoing photon therapy will be treated with an image-guided conformal radiation technique [three-dimensional conformal radiation therapy (3D-CRT), intensity-modulated radiation therapy (IMRT)]. For proton radiotherapy, patient positioning will be performed based on orthogonal X-ray and correction for the bony anatomy. Target coverage and dose to organs at risk will be assessed by at least one physician and one physicist prior to the start of treatment. Constraints will be according to current clinical guidelines based on QUANTEC. All patients who are treated with protons receive therapy with passively scattered or actively scanned protons.

Patients will be treated either with an accelerated irradiation schedule or with conventional fractionation (7 or 5 fractions per week, 2 Gy fractions, respectively). The mediastinal target volume will be defined according to clinical guidelines and irradiated up to a total dose of 50 Gy. In addition, boost irradiation of 10 Gy (2 Gy single dose) will be applied in cases of a partial resection (R1 situation) or extracapsular tumor spread (ECE) (total dose 60 Gy). For patients with an R2 situation or a detected recurrence postoperatively/after adjuvant chemotherapy (“R” stratum), the boost dose is escalated to 16 Gy. In this group, the total dose of thus 66 Gy is administered with standard fractionation (5 fractions/week).

Accelerated radiotherapy is performed by application of a second fraction on 2 (not consecutive) days every week and/or an additional irradiation at weekends. It is mandatory to have an interfraction interval of at least 6, preferably even 8 hours.

## Discussion

The presented phase II randomized trial evaluates for the first time if an accelerated postoperative irradiation schedule in NSCLC patients results in higher local tumor control rates compared to conventional fractionation. Several arguments support our hypothesis: a time factor, i.e., an impaired local tumor control after longer OTT of radiotherapy has been shown for primary radiotherapy schedules in NSCLC [14–18]; repopulation as the main reason for this time factor has been shown to be potentially induced by chemotherapy and this in turn is applied upfront to radiotherapy in the adjuvant situation [11]; our own retrospective data show impaired local tumor control rates after longer versus shorter overall treatment times of adjuvant radiotherapy [13]. In detail, a significant correlation between overall radiation treatment time and overall survival (61% vs. 36% at 2 years, *p* = 0.001), relapse-free (90% vs. 51% at 2 years, *p* = 0.002) and metastases-free survival (54% vs. 43% at 2 years, *p* = 0.015) was found in this study.

For acceleration of radiotherapy, the present study uses standard doses of 2 Gy per fraction and increases the radiation dose per week to 14 Gy. This is in contrast to previous acceleration trials on primary radiotherapy, where accelerated hyperfractionated schedules have been used with up to 3 irradiation fractions of 1.5 Gy per day on 5–7 days per week [16, 17]. The reason for the additional use of hyperfractionation was that extremely short treatment schedules have been used with a high dose-intensity, i.e., high dose per day or per week. Hyperfractionation instead of the application of the whole daily dose in one fraction leads here to better sparing of normal tissues due to repair and recovery between the fractions. Based on our own retrospective data the present trial uses a more moderate acceleration schedule. The application of 2 Gy per fraction is not only easier to apply in daily workflows of radiotherapy departments, it is also coming closer to recent radiotherapy schedules applied in dose escalation trials that are using doses of > 2 Gy per fraction [21].

In addition, further optimization of postoperative irradiation in NSCLC patients may include the use of proton or heavier ion radiotherapy, which may enable better sparing of normal tissues but would hardly be applicable in hyperfractionated schedules. Therefore, the influence of proton therapy on early and late toxicity in the adjuvant situation in NSCLC patients will be investigated in this trial.

### Trial status

Patient accrual started in November 2014 and is currently ongoing. At present, 14 centers participate in this study (Germany and Poland).

## Additional files

Additional file 1: SPIRIT 2013 Checklist: Recommended items to address in a clinical trial protocol and related documents*. (DOC 122 kb)

Additional file 2: SPIRIT figure. (DOC 49 kb)

## Abbreviations

3D-CRT: Three-dimensional conformal radiation therapy
4D-CT: Four-dimensional computed tomography
CT: Computed tomography
CTC-AE: Common Terminology Criteria for Adverse Events
CTV: Clinical target volume
FDG: Fluorodeoxyglucose
Gy: Gray
IMRT: Intensity-modulated radiation therapy
MRI: Magnetic resonance imaging
NSCL: Non-small-cell lung carcinoma
OTT: overall treatment time
PET: Positron emission tomography
PORT: Postoperative radiotherapy
