# Supplementary material for: PORTAF – postoperative radiotherapy of non-small cell lung cancer: accelerated versus conventional fractionation – study protocol for a randomized controlled trial
Source: Trials. 2017 Dec 20;18:608. doi: 10.1186/s13063-017-2346-0 (PMC5738814; doi:10.1186/s13063-017-2346-0)
Supplement: Supplementary file 2 — SPIRIT figure. (DOC 49 kb) [file 13063_2017_2346_MOESM2_ESM.doc]

|  | **STUDY PERIOD** | | | | | | | |
| --- | --- | --- | --- | --- | --- | --- | --- | --- |
|  | **Enrolment** | **Allocation** | **Post-allocation** | | | | | **Close-out** |
| **TIMEPOINT**** | ***-t_1_*** | **0** | **1^st^ fx** | **last fx** | **6 weeks after fx** | **every 3 months until 3 years after fx** | **every 6 months until 5 years after fx** | **5 years after fx** |
| **ENROLMENT:** |  |  |  |  |  |  |  |  |
| **Eligibility screen** | X |  |  |  |  |  |  |  |
| **Informed consent** | X |  |  |  |  |  |  |  |
| **Quality of life questionare** | X |  |  |  |  |  |  |  |
| **Allocation** |  | X |  |  |  |  |  |  |
| **INTERVENTIONS:** |  |  |  |  |  |  |  |  |
| **Conventional fractionation** |  |  |  |  |  |  |  |  |
| **Accelerated fractionation** |  |  |  |  |  |  |  |  |
| **“R” stratum** |  |  |  |  |  |  |  |  |
| **ASSESSMENTS:** |  |  |  |  |  |  |  |  |
| **Clinical examination** | X |  | X | X | X | X | X | X |
| **Imaging** | X |  |  |  | X | X | X | X |
| **Assessment of side effects** |  |  | X | X | X | X | X | X |
| **Quality of life questionnaire** | X |  |  | X | X | X | X | X |
